# Met-enkephalin and other opioid peptides in the stress response of chickens: lessons from laboratory animals and livestock

**DOI:** 10.3389/fphys.2026.1756240

**Published:** 2026-02-26

**Authors:** Krystyna Pierzchała-Koziec, Colin G. Scanes

**Affiliations:** 1 University of Agriculture in Krakow, Krakow, Poland; 2 University of Wisconsin Milwaukee, Milwaukee, WI, United States

**Keywords:** chicken, dynorphin, extended enkephalin, Leu-enkephalin, Met-enkephalin, stress

## 1 Introduction

Endogenous opioid peptides and their receptors are involved in the regulation of many physiological processes, including nociception, analgesia, respiration, cardiovascular and gastrointestinal system activity, as well as nervous, endocrine, and immune functions. Opioid peptides, forming the family of enkephalins, dynorphins, and endorphins, are synthesized as large peptides (precursors) named preproenkephalin, preprodynorphin, and proopiomelanocortin, respectively.

### 1.1 Genes encoding opioid peptides

Opioid peptides from all families are encoded by four genes:

A. Proenkephalin (*PENK*) encodes a specific protein:
  a. Met-enkephalin (YGGFM),
  b. Leu-enkephalin (YGGFL),
  c. Extended Met-enkephalin (YGGFMXX).
B. Prodynorphin (*PDYN*) encodes the following:
  a. Dynorphins A and B
  b. α- and β-neoendorphins.
C. Proopiomelanocortin (*POMC*) encodes β endorphin together with adrenocorticotropic hormone (ACTH).
D. Pronociceptin (*PNOC*) encodes nociceptin.

### 1.2 Opioid receptors

Opioid peptides belong to the G-protein-coupled receptor superfamily and act by binding to opioid receptors localized in the brain and peripheral tissues, specifically the following:

A. Delta opioid receptors (DORs), binding Met- and Leu-enkephalin,
B. Kappa opioid receptors (KORs), binding dynorphins A and B,
C. Mu opioid receptors (MORs), binding β-endorphin,
D. Recently, the nonopioid orphanin FQ/nociceptin (NOP) receptor was included; it binds nociceptin.

Opioid properties were broadly searched as important modulators of hypothalamic–pituitary–adrenal (HPA) axis activity, particularly during stress responses. Endorphin- and enkephalin-producing neurons are present in the paraventricular nucleus and the median eminence and modulate adrenocorticotropic hormone (ACTH)-controlling neurons (Van'T Veer et al., 2012).

The present communication focuses on Met-enkephalin in chickens, the effect of stress on Met-enkephalin physiology, insights gained from laboratory animals and livestock, and open questions on opioid peptides.

## 2 Loci for Met-enkephalin synthesis and release

Met-enkephalin is found in multiple tissues of rats, including the anterior pituitary gland, neurointermediate lobe of the pituitary gland, adrenal gland, hypothalamus, heart, lung, spleen, liver, seminal vesicle, vas deferens, kidney, bladder detrusor, and duodenum, with the highest concentration in the neurointermediate lobe (Kolta et al., 1992). Similarly, in chickens, Met-enkephalin is synthesized in the hypothalamus, anterior pituitary gland, adrenal glands, duodenum, proventriculus, and crop (Scanes and Pierzchala-Koziec, 2024a; Scanes and Pierzchala-Koziec, 2024b).

## 3 Met-enkephalin and stress

Stress (imposition of mechanical restraint) in rats is followed rapidly by increases in plasma concentrations of native (pentapeptide) Met-enkephalin (Pierzchała and Van Loon, 1990). Concentrations of Met-enkephalin are also elevated in lambs isolated from other sheep, including dams (Pierzchała-Koziec et al., 2018; 2019). In chickens, both plasma concentrations of pentapeptide Met-enkephalin and PENK expression are elevated in young chickens subjected to restraint stress (Scanes et al., 2024). There are also effects of other stresses on plasma concentrations of pentapeptide Met-enkephalin and PENK expression. For instance, withholding water was accompanied by depressed concentrations of Met-enkephalin in both the anterior pituitary and adrenal glands, together with increased PENK expression in the same organs (Scanes and Pierzchala-Koziec, 2024a). Moreover, there are decreased plasma concentrations of Met -enkephalin in chickens deprived of feed (Scanes and Pierzchala-Koziec, 2024a). There is increasing evidence that Met-enkephalin plays a role in the immune system (Zhao et al., 2016; Tian et al., 2018; 2024; Wang et al., 2018). The relationships between Met-enkephalin and immune functioning in chickens remain unclear.

## 4 Control of pentapeptide Met-enkephalin release

The neurotransmitter acetylcholine plays an important role in regulating the release and synthesis of the native pentapeptide Met-enkephalin. The release of Met-enkephalin from the adrenal glands is under cholinergic control, as evidenced by with the nicotinic agonist nicotine, which increases concentrations of both native Met- and Leu-enkephalin in the adrenal medulla and other tissues in rats (Van Loon et al., 1991; Pierzchała-Koziec and Van Loon, 1994). Moreover, *in vitro* Met-enkephalin release and PENK gene expression have been observed in the hypothalamus, anterior pituitary gland, adrenal glands, crop, proventriculus, and duodenum in chickens (Scanes et al., 2024; 2025). Intestinal explants, at least, exhibit shifts in both PENK gene expression and Met-enkephalin release in the presence of both nicotinic and muscarinic cholinergic antagonists (Scanes et al., 2025).

Opioids downregulate the Met-enkephalin system. The classical opioid morphine depresses plasma concentrations of Met-enkephalin and PENK expression in both the anterior pituitary and adrenal glands in young chickens (Scanes and Pierzchala-Koziec, 2024b). Moreover, the effects of restraint stress are attenuated by the administration of the opioid antagonist naltrexone (Scanes et al., 2024).

## 5 Total immuno-reactive Met-enkephalin in plasma and tissues

Stress in rats is followed rapidly by shifts in plasma concentrations of total Met-enkephalin (Pierzchała and Van Loon, 1990), with the latter being generated by enzymatic hydrolysis of plasma proteins. There are analogous changes in plasma concentrations of total Met-enkephalin in lambs isolated from their dams (Pierzchała-Koziec et al., 2018; 2019). It remains unclear what total Met-enkephalin signifies. Possible explanations include the following:

1. Proenkephalin or peptides larger than the pentapeptide Met-enkephalin that are derived from proenkephalin but lack immuno-reactivity in the native Met-enkephalin radioimmunoassay.
2. Met-enkephalin binding to proteins in the circulation and/or secretory granules.
3. A combination of possibilities 1 and 2.

**FIGURE 1:**
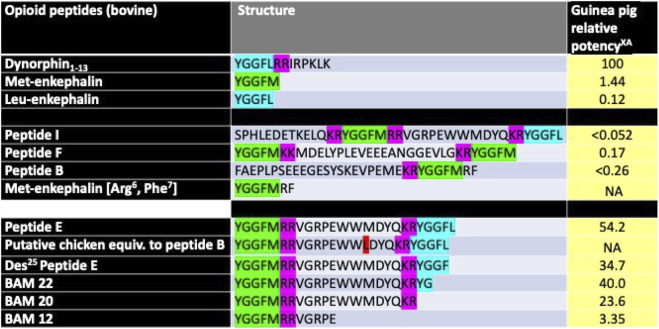
Structure of selected opioid peptides isolated from cattle adrenal glands together with putative chicken homologs and biological activities in a presumed KOR assay. ^X^Relative to dynorphin_1-13_ as 100. ^A^Calculated from IC_50_s from Goldstein et al., 1979; Kilpatrick et al., 1981. NA, not available [Key: pink–dibasic amino-acid residues, green–Met-enkephalin residues, blue–Leu-enkephalin residues, red–difference between chicken and bovine sequences).

Multiple peptides are derived from proenkephalin in the bovine and presumably chicken adrenal glands, including extended Met-enkephalin, Met-enkephalin [Arg^6^ and Phe^7^] peptides B, E, F, and I, and BAM 22, 20, and 12 (Stern et al., 1979; 1981), with different activities (Figure 1).

## 6 Other opioid peptides and stress

To the best of our knowledge, there are no reports of Leu-enkephalin, dynorphins A and B together, and α- and β-neoendorphins in birds. There are few reports of dynorphins even in humans. Similarly, there is only a single report of circulating concentrations of β endorphin in chickens, in which the molecular forms of β-endorphin were examined (Hylka and Thommes, 1991). In addition, plasma concentrations of both ACTH and β-endorphin increase in response to stressors, such as exposure to ether or administration of lipopolysaccharide, in domestic geese (Barna et al., 1998).

## 7 Discussion and conclusions

The physiological relevance of circulating Met-enkephalin and other endogenous opioid peptides in birds remains poorly understood. To better document the physiology of Met-enkephalin acting as a hormone, studies on its circulating forms and their regulation are essential. Thus, during the past few years, we have measured immunoreactive Met-enkephalin in plasma and tissues to characterize the large circulating forms of peptidase-derivable Met-enkephalin and define, in hens, the physiological regulation of plasma responses of free Met-enkephalin (five amino acids) and the extended form of Met-enkephalin to psychological stress. Similar to rats (Pierzchala and Van Loon, 1990), restraint- or crowding-induced stress elicited biphasic responses of Met-enkephalin (Scanes et al., 2024; Scanes and Pierzchala-Koziec, 2024a).

Restraint stress in rats increased plasma native Met-enkephalin, which is in parallel with the increases in plasma epinephrine and norepinephrine. Thereafter, there was a divergence in the plasma concentrations of Met-enkephalin and catecholamines during the period of restraint stress. Plasma Met-enkephalin showed a biphasic response to 30 min of restraint: increasing at 1 and 30 min of stress; in contrast, catecholamines increased only at 1–3 min of restraint. It seems probable that the brief duration of the initial peak of plasma Met-enkephalin induced by restraint stress results from a central nervous system regulatory mechanism (interaction with the sympathetic nervous system) rather than from a limitation in Met-enkephalin pool size since the more severe stress of immobilization produced a prolonged elevation of plasma Met-enkephalin (Pierzchała-Koziec and Van Loon, 1994). In hens, depletion of peripheral catecholamine sources did not decrease Met-enkephalin responses to restraint stress but may indicate the involvement of additional regulators of opioid synthesis and release, apart from catecholamines, such as acetylcholine, insulin, and ghrelin.

This short review on the role of Met-enkephalin in modulating stress responses showed that, despite extensive scientific research, several questions regarding opioid peptides in birds remain unresolved:

1. Met-enkephalin is produced by multiple organs. It remains unclear which, if any, are the major sources of circulating Met-enkephalin.
2. It is also unclear to what extent, if any, erythrocytes, leukocytes, and/or thrombocytes release or degrade Met-enkephalin.
3. It remains uncertain whether Met-enkephalin exerts its effects via paracrine and endocrine mechanisms.
4. Plasma concentrations of total Met-enkephalin (generated by enzymatic hydrolysis of plasma proteins) greatly exceed those of native pentapeptide Met-enkephalin. It remains unclear the extent to which total Met-enkephalin reflects larger cleavage products of proenkephalin and/or binding of Met-enkephalin to plasma proteins.
5. There is a series of extended Met-enkephalin peptides in cattle. It remains unclear whether these peptides are also found in chickens and other birds, whether they are secreted in response to stimuli, and what their physiological actions are.
6. There are no published reports on the effects of stress and other physiological interventions on circulating concentrations of Leu-enkephalin, prodynorphin-derived peptides, or nociceptin in chickens or other birds.
7. There are few published reports (<5) on the effects of stress and other physiological interventions on circulating concentrations of β-endorphin in chickens or other birds.

Answers to the abovementioned questions will clarify the role of endogenous opioids in stress and may facilitate opioid peptides being indicators of stress/failures in welfare. Moreover, it is speculated that research on opioid peptides will provide new bases for dissecting the multiple facets of stress and the responses to these.
